# High Neutrophil-To-Lymphocyte Ratio Is an Independent Risk Factor for End Stage Renal Diseases in IgA Nephropathy

**DOI:** 10.3389/fimmu.2021.700224

**Published:** 2021-08-12

**Authors:** Siqing Wang, Lingqiu Dong, Gaiqin Pei, Zheng Jiang, Aiya Qin, Jiaxing Tan, Yi Tang, Wei Qin

**Affiliations:** ^1^West China School of Medicine, Sichuan University, Chengdu, China; ^2^Division of Nephrology, Department of Medicine, West China Hospital of Sichuan University, Chengdu, China; ^3^Division of Rehabilitation, Department of Medicine, West China Hospital of Sichuan University, Chengdu, China; ^4^Key Laboratory of Rehabilitation Medicine in Sichuan Province, Sichuan University, Chengdu, China

**Keywords:** IgA nephropathy, neutrophil-to-lymphocyte ratio (NLR), pathologic lesions, renal progression, renal prognosis

## Abstract

**Background:**

Complex factors are involved in the development and progression of immunoglobulin A nephropathy (IgAN), a common primary glomerulonephritis worldwide. Autoimmunity and inflammation have been considered to be the basic mechanisms; however, the exact pathogenesis remains unclear. As a novel marker of inflammation, the neutrophil-to-lymphocyte ratio (NLR) has been studied in various diseases. Whether the NLR can predict the renal outcome of patients with IgAN remains unclear. We evaluated the relationships between the NLR and renal function, pathologic lesions, renal progression, and prognosis in patients with IgAN.

**Methods:**

This retrospective study involved 966 patients with biopsy-proven IgAN. They were divided into two groups based on the cut-off value of the NLR: the high group (NLR ≥ 2.67, n = 384) and the low group (NLR < 2.67, n = 582). The endpoint was end-stage renal disease [estimated glomerular filtration rate (eGFR) of <15 mL/min/1.73 m^2^ or performance of renal replacement therapy]. A correlation test was conducted to explore the relationship between the NLR and other important parameters (eGFR, serum creatinine, proteinuria, hypertension and renal pathologic lesions). The predictive value was determined by the area under the receiver operating characteristics curve (AUROC). Kaplan–Meier and Cox proportional hazards analyses were performed to evaluate renal progression and prognosis.

**Results:**

The NLR had the highest AUROC, which was 0.633 (p < 0.001). The correlation test revealed that the NLR was positively correlated with serum creatinine (r = 0.127, p < 0.001) and 24-hour urine protein (r = 0.18, p < 0.001) and negatively correlated with eGFR (r = 0.14, p < 0.001). Patients with IgAN who had a high NLR were more likely to have hypertension (p = 0.003). Multivariate Cox regression analysis indicated that a high NLR was an independent risk factor for IgAN even after adjustment for important clinical and pathological parameters (p = 0.043, HR = 1.74, 95%CI: 1.02-2.97). Kaplan–Meier analysis showed that a high NLR was significantly associated with the renal prognosis of patients with IgAN (p < 0.001), especially patients with stage 3 to 4 chronic kidney disease (p = 0.028) or 24-hour urine protein of >1 g/day (p < 0.001).

**Conclusion:**

An elevated NLR affects the renal progression and prognosis in patients with IgAN and could be a marker for evaluation of renal function and pathologic lesions.

## Introduction

Immunoglobulin A nephropathy (IgAN) is one of the most common types of primary glomerulonephritis worldwide (1). Studies showed that about 20-40% IgAN patients reached end-stage renal disease (ESRD) 10 to 20 years after the onset of IgAN, costing considerable socioeconomic resources (2). Thus, the identification of patients with IgAN who are at high risk of progressive reduction of renal function is worthwhile.

Blood count is commonly used in daily clinical practice. Neutrophils (NEs) and lymphocytes (Lys) play important roles in the innate and adaptive immune systems. Chronic inflammation reportedly participates in the pathogenesis of IgAN (3). The peripheral NE-to-LY ratio (NLR) has been recognized as a novel inflammatory marker in various diseases such as atherosclerosis (4), hepatocellular carcinoma (5), type 2 diabetes mellitus, and diabetic nephropathy (6). However, very few studies addressed the relationship between the NLR and IgAN (7, 8), which reported conflicting results. Moreover, the precise relationship between the NLR and clinical and pathologic parameters has not been investigated. Therefore, whether the NLR is a risk factor for ESRD in patients with IgAN remains controversial.

We performed the present retrospective study of 966 patients with IgAN for a median follow-up period of 58 months to provide more evidence about the relationship between the NLR and renal function, pathologic lesions, renal progression, and prognosis.

## Materials and Methods

### Patients

**Figure 1:**
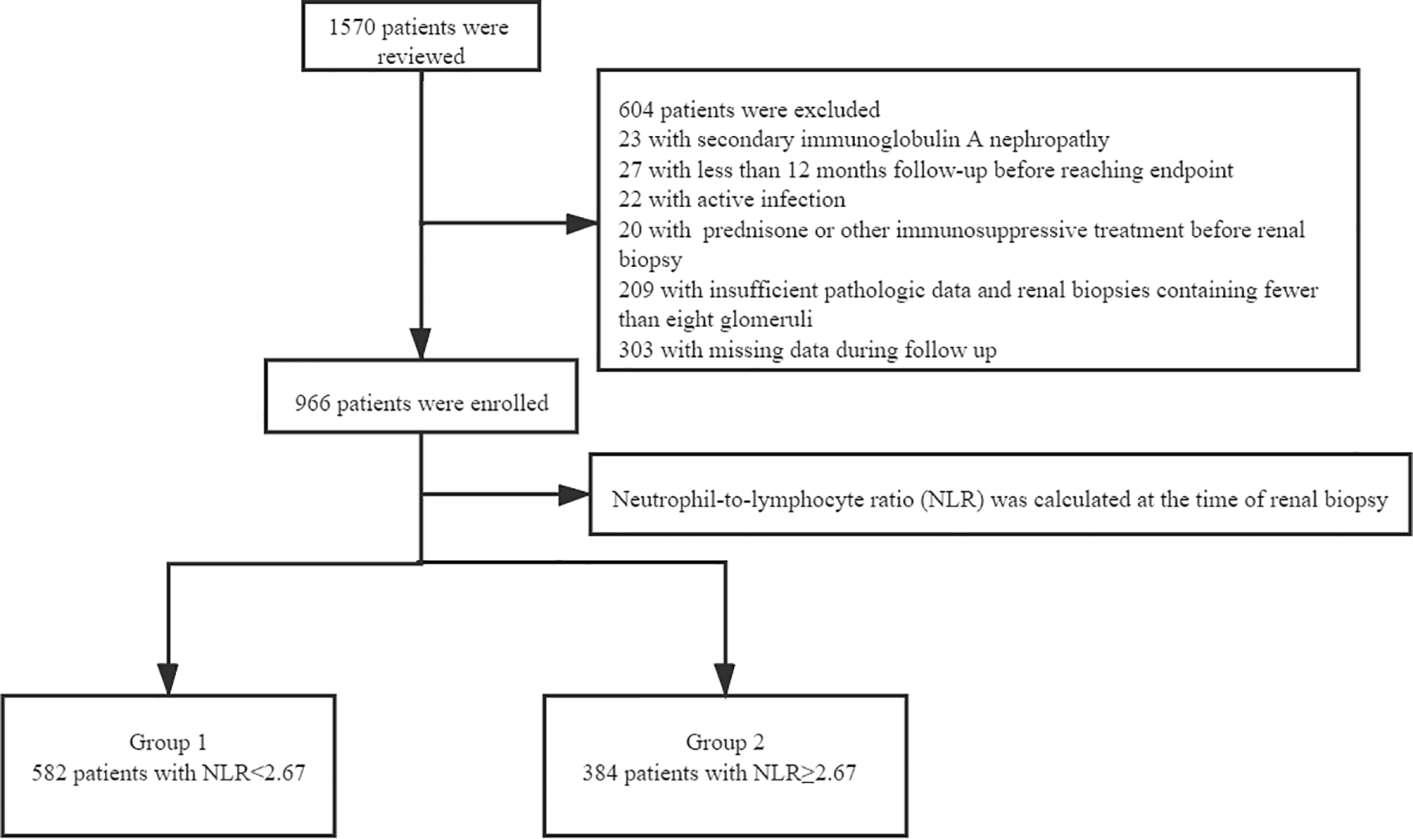
The flow chart of excluded patients.

This study involved 1570 adults with renal biopsy-proven IgAN at West China Hospital of Sichuan University from January 2009 to December 2018. The diagnosis of IgAN was achieved by renal biopsy, which showed the predominance of IgA deposits in the glomerular mesangium, either alone or with IgG, IgM, or complement C3 (1). We excluded patients with systemic diseases (including but not limited to systemic lupus erythematosus, diabetes mellitus, Henoch–Schönlein purpura, and liver cirrhosis), active infection, treatment with prednisone or other immunosuppressive agents before renal biopsy, other diseases or treatment agents that could impact the NLR, insufficient pathologic data, renal biopsies containing fewer than eight glomeruli, or missing data during follow-up. All patients were followed up for at least 12 months before reaching the study endpoint (**Figure 1**). Written informed consent for participation in the study was obtained from all patients. The study was approved by the Ethics Committee of West China Hospital of Sichuan University (2019-33), and all methods were carried out in accordance with relevant guidelines and regulations. After renal biopsy, all patients were followed up at least every 1 to 3 months at the outpatient department of West China Hospital or by telephone.

### Clinical Data

Demographics (sex, age) and clinical data [serum creatinine, hemoglobin, PLT count, NE count, WBC count, LY count, serum albumin, total cholesterol, uric acid, 24-hour urine protein, and estimated glomerular filtration rate (eGFR)] were collected at the time of renal biopsy and at the follow-up visit. Hypertension was defined as blood pressure of >140/90 mmHg or the use of antihypertensive agents. eGFR was calculated using the CKD-EPI equation. Anemia was defined as a hemoglobin concentration of <120 g/L in men and <110 g/L in women. Hyperuricemia was defined as a uric acid concentration of >420 µmol/L in men and >360 µmol/L in women.

### Pathological Data

Renal biopsy samples were evaluated by light microscopy (hematoxylin–eosin, periodic acid–Schiff, Masson, and periodic acid–Schiff/methenamine staining), immunofluorescence (IgA, IgG, IgM, C3, C4, and C1q), and electron microscopy by experienced pathologists and nephrologists according to the Oxford classification of IgAN: mesangial hypercellularity (M0/M1), endocapillary hypercellularity (E0/E1), segmental glomerulosclerosis (S0/S1), tubular atrophy/interstitial fibrosis (T0/T1/T2), and cellular or fibrocellular crescents (C0/C1/C2) (9).

### Treatment Data and Endpoint

All patients were divided into a supportive treatment group and a prednisone or other immunosuppressive agents group according to their treatment after renal biopsy. Besides, the treatment plan was selected according to the guidelines and renal pathological lesions. The study endpoint was ESRD, which was defined as an eGFR of <15 mL/min/1.73 m^2^ or performance of renal replacement therapy.

### Evaluation of Predictive Value of Different Factors

The discriminatory power of various predictive factors for development of ESRD was tested by the area under the receiver operating characteristic curve (AUROC), and the optimal cut-off point of the NLR was obtained by calculating the Youden index. The Youden index is a method of evaluating the authenticity of a screening test, which represents the total ability of the screening method to detect true patients and non-patients. A higher index is associated with a better effect and greater authenticity of the screening test. The NLR that corresponded to the maximum Youden index was then determined to be the optimal cut-off NLR in this study.

### Statistical Analysis

All statistical analyses were performed using IBM SPSS software, version 22.0 (IBM Corp., Armonk, NY, USA). Continuous variables are expressed as mean ± standard deviation or median with interquartile range and were analyzed by an unpaired t-test or the Kruskal–Wallis H test or the nonparametric Mann–Whitney U test as appropriate, for normally and non-normally distributed variables (the Shapiro-Wilk test). Categorical data were analyzed using the chi-square test and are presented as frequency (percentage). The relationships between the NLR and other important parameters (eGFR, serum creatinine, proteinuria, hypertension and renal pathologic lesions) were explored by a correlation test. Kaplan–Meier and Cox proportional hazards analyses were performed to evaluate renal progression and prognosis. Results are expressed as hazard ratios (HRs) and 95% confidence intervals (CIs). Statistical significance was considered if p < 0.05.

## Results

### Predictive Value of NLR for ESRD

**Figure 2:**
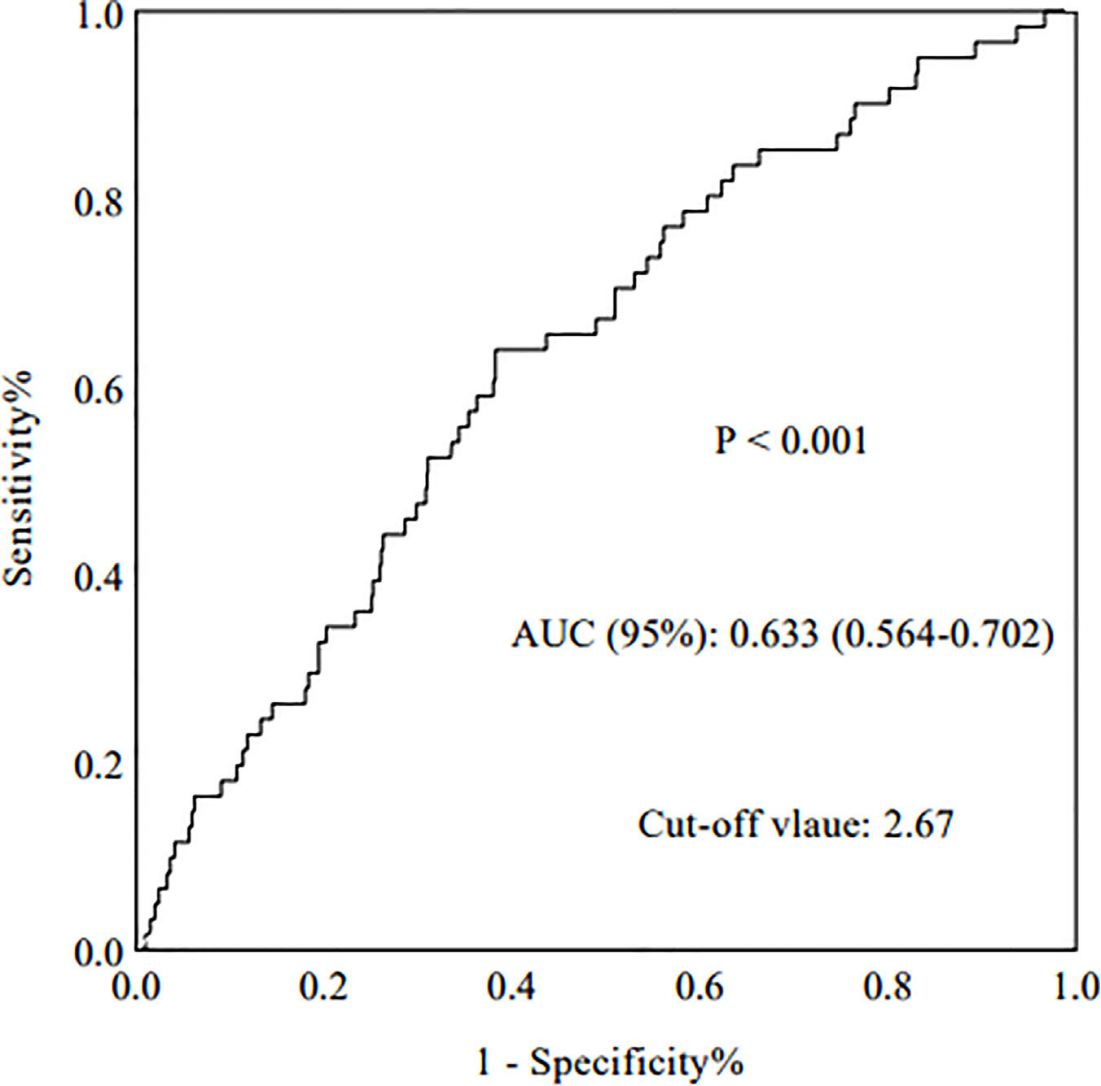
The areas under the ROC curves (AUCs) of NLR.

The AUROC was analyzed to determine the predictive value of relevant indictors. Compared with routine inflammatory factors [WBCs, PLTs, LYs, PLT-to-LY ratio (PLR), and NEs], the NLR had the highest AUROC (0.633) (p < 0.001, 95%CI: 0.564-0.702) (**Figure 2**). The AUROC values for WBCs, PLTs, LYs, the PLR, and NEs were 0.523, 0.537, 0.389, 0.607, and 0.559, respectively (**Table 1**).

**Table 1:** The diagnostic accuracy of various prediction factors for development of ESRD in IgAN patients.

Parameters	P value	AUC (95%)
NLR	<0.001	0.633 (0.564-0.702)
PLR	0.005	0.607 (0.537-0.677)
WBC (×10^9^/L)	0.550	0.523 (0.447-0.599)
PLT (×10^9^/L)	0.328	0.537 (0.462-0.613)
LY (×10^9^/L)	0.004	0.389 (0.317-0.461)
NE (×10^9^/L)	0.122	0.559 (0.487-0.632)

PLT, platelet counts; NE, neutrophils counts; LY, lymphocytes counts; PLR, platelet‐to‐lymphocyte ratio; WBC, white blood cell counts; NLR, Neutrophil-to-lymphocyte ratio.

### Demographic and Clinicopathological Characteristics

The baseline data of the 966 patients are shown in **Table 2**. All patients enrolled in this study were divided into two groups according to their NLR at the time of renal biopsy. ROC analysis revealed that the optimal cut-off NLR with which to predict the progression of ESRD in patients with IgAN was 2.67, with a sensitivity and specificity of 64% and 62%, respectively. Next, all patients were distributed into a high group (NLR ≥ 2.67) and low group (NLR < 2.67). The median follow-up time was 58.67 months, the median age of all patients was 35 (26,42) years, and 45.9% of the patients were male. Compared with the low group, patients in the high NLR group had a higher incidence of proteinuria (p < 0.001), higher incidence of hypertension (p = 0.003), and worse renal function. The median eGFR in the high-NLR and low-NLR groups was 87.05 and 96.23 mL/min/1.73 m^2^, respectively. Based on the Oxford classification of IgAN, we found that patients with a high NLR always had segmental glomerulosclerosis (p < 0.001) and tubular atrophy/interstitial fibrosis (p < 0.001).

**Table 2:** Demographic and clinicopathological characteristics of 966 IgAN patients.

Parameters	Group 1 (NLR < 2.67) n = 582	Group 2 (NLR ≥ 2.67) n = 384	P value
Male	269 (46.2%)	174 (45.3%)	0.782
Age (years)	32 (25,41)	34 (26,44)	0.024
Hypertension (%)	135 (23.2%)	122 (31.8%)	0.003
Smoking (%)	101 (17.4%)	71 (18.5%)	0.652
Anemia (%)	167 (28.7%)	124 (32.3%)	0.233
PLT (×10^9^/L)	185 (147,229)	190 (150,236)	0.296
NE (×10^9^/L)	3.71 (2.93,4.48)	5.45 (4.38,6.87)	<0.001
WBC (×10^9^/L)	6.25 (5.16,7.41)	7.56 (6.29,9.52)	<0.001
LY (×10^9^/L)	1.98 (1.66,2.37)	1.45 (1.2,1.75)	<0.001
PLR	95.3 (72.8,118.1)	132.1 (102.3,162.2)	<0.001
NLR	1.92 (1.56,2.26)	3.49 (3.02,4.62)	<0.001
Proteinuria (g/d)	1.17 (0.7,2.3)	2 (1,3)	<0.001
Proteinuria classification			<0.001
24h-proteinuria<1g/d	263 (45.2%)	120 (31.3%)	
24h-proteinuria≥1g/d	319 (54.8%)	264 (68.7%)	
URBC (/HP)	19 (6,74)	19 (6,62)	0.9257
ALB (g/L)	40.7 (37.3,43.5)	39.6 (34.8,43.1)	0.002
Cr (umol/L)	80.55 (64,103.93)	88.9 (67.25, 121)	0.001
eGFR (mL/min/1.73 m^2^)	96.23 (72.97,119.32)	87.05 (55.66,113.21)	<0.001
CKD stages			<0.001
Stage 1	332 (57%)	186 (48.4%)	
Stage 2	157 (27%)	89 (23.2%)	
Stage 3	80 (13.8%)	86 (22.4%)	
Stage 4	13 (2.2%)	23 (6%)	
Hyperuricemia (%)	220 (37.8%)	163 (42.4%)	0.148
Hypertriglyceridemia (%)	117 (20.1%)	93 (24.2%)	0.129
Hypercholesterolemia (%)	65 (11.2%)	77 (20.1%)	<0.001
M1	431 (74%)	300 (78.1%)	0.174
E1	21 (3.6%)	20 (5.2%)	0.227
S1	332 (57%)	262 (68.2%)	<0.001
T1/2	92 (15.8%)	103 (26.8%)	<0.001
C1/2	127 (21.8%)	102 (26.5%)	0.195
Treatment			<0.001
Support treatment (%)	268 (46%)	129 (33.6%)	
Prednisone or other immunosuppressive agents (%)	314 (54%)	255 (66.4%)	

PLT, platelet counts; NE, neutrophils counts; LY, lymphocytes counts; PLR, platelet‐to‐lymphocyte ratio; WBC, white blood cell counts; NLR, Neutrophil-to-lymphocyte ratio; URBC, urinary red blood cell counts; ALB, albumin; Cr, creatinine; eGFR, estimated glomerular filtration rate; M, mesangial proliferation; E, endocapillary proliferation; S, segmental sclerosis; T, tubular atrophy/interstitial fibrosis; C, crescents; CKD, chronic kidney disease.

### Correlation of NLR With Clinical Parameters and Pathologic Lesions

Correlation analyses were performed to elucidate the relationship between the NLR and important clinicopathological factors. Our results showed that the NLR was significantly correlated with the eGFR (r = −0.14, p < 0.001), serum creatinine (r = 0.127, p < 0.001), and proteinuria (r = 0.18, p < 0.001). Additionally, patients with IgAN who had an NLR of ≥2.67 were likely to have hypertension [odds ratio (OR), 1.54; 95% CI, 1.16–2.06; p = 0.003]. Moreover, patients with IgAN who had a high NLR tended to have pathologic lesions with segmental glomerulosclerosis (OR, 1.61; 95% CI, 1.23–2.12; p < 0.001), tubular atrophy/interstitial fibrosis (OR, 1.95; 95% CI, 1.42–2.68; p < 0.001), and cellular or fibrocellular crescents (OR, 1.30; 95% CI, 0.96–1.75; p < 0.001).

### NLR as an Independent Risk Factor for Progression of IgAN to ESRD

Univariate Cox regression analysis revealed that a high NLR was a risk factor for progression to ESRD (HR, 2.68; 95% CI, 1.60–4.50; p < 0.001). Moreover, multivariate Cox regression analysis indicated that a high NLR was an independent risk factor for renal progression after adjustment for pathologic lesions and important clinical parameters, such as the Oxford MEST-C score, sex, age, hypertension, anemia, serum albumin concentration of <30 g/L, hypertriglyceridemia, 24-hour urine protein, eGFR of <30 mL/min/1.73 m^2^, hyperuricemia, smoking status, hypercholesterolemia, and PLT-to-NE ratio (HR, 1.74; 95% CI, 0.98–3.05; p = 0.043) (**Table 3**).

**Table 3:** Univariate and multivariate Cox proportional hazard model for the renal outcome in 966 IgAN patients.

Parameter	Univariate			Multivariate		
	HR	95%CI	P value	HR	95%CI	P value
High NLR	2.68	1.60-4.50	<0.001	1.74	0.98-3.05	0.043
Male	2.23	1.32-3.76	0.003	1.02	0.51-2.06	0.947
Age	1.00	0.98-1.02	0.993	1.00	0.97-1.03	0.831
Oxford Classification						
M1/M0	4.51	1.64-12.46	0.004	2.54	0.89-7.26	0.081
E1/E0	1.93	0.77-4.81	0.160	1.37	0.48-3.95	0.557
S1/S0	2.82	1.53-5.23	0.001	1.37	0.67-2.80	0.397
T1-2/T0	14.52	8.10-26.04	<0.001	4.95	2.49-9.84	<0.001
C1-2/C0	1.92	1.15-3.21	0.013	1.24	0.69-2.26	0.476
Treatment of prednisone or immunosuppressive agents	0.76	0.46-1.25	0.280	0.37	0.21-0.66	0.001
Hypertension	3.93	2.36-6.55	<0.001	1.21	0.64-2.29	0.555
Serum albumin<30g/L	2.04	1.06-3.92	0.032	0.84	0.32-2.26	0.732
Anemia	4.29	2.54-7.24	<0.001	2.34	1.30-4.22	0.005
Hypertriglyceridemia	1.91	1.12-3.25	0.018	0.86	0.46-1.60	0.635
24h-proteinuria	1.11	1.06-1.17	<0.001	1.10	1.01-1.21	0.035
eGFR<30mL/min/1.73m2	22.65	13.30-38.57	<0.001	4.28	2.12-8.64	<0.001
Hyperuricemia	5.63	3.14-10.11	<0.001	2.92	1.47-5.78	0.002
PLR	1.00	0.99-1.01	0.574	1.00	0.99-1.01	0.674
Smoking	2.06	1.19-3.57	0.010	1.66	0.78-3.57	0.191
Hypercholesterolemia	1.57	0.85-2.89	0.151	1.26	0.54-2.94	0.594

PLR, platelet‐to‐lymphocyte ratio; NLR, Neutrophil-to-lymphocyte ratio; eGFR, estimated glomerular filtration rate; M, mesangial proliferation; E, endocapillary proliferation; S, segmental sclerosis; T, tubular atrophy/interstitial fibrosis; C, crescents.

### Use of NLR to Predict Treatment Response and Renal Prognosis

**Figure 3:**
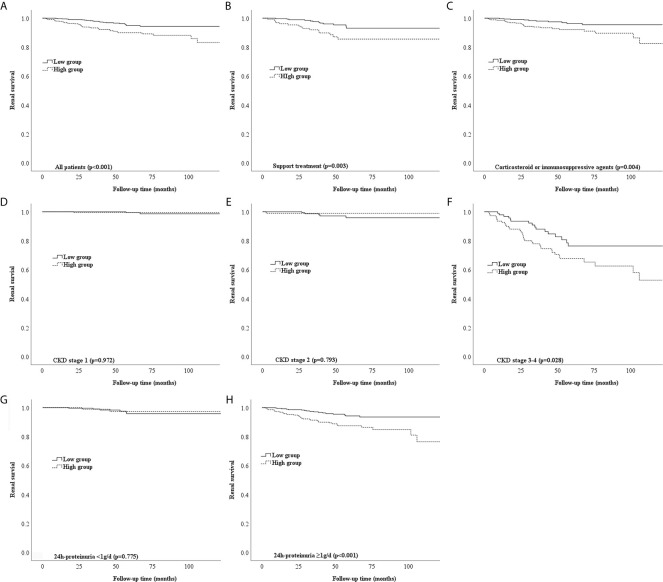
Different types of Kaplan-Meier analysis for the endpoint of ESRD. **(A)** Kaplan-Meier analysis for all patients to the endpoint of ESRD. **(B,C)** Kaplan-Meier analysis for patients with different treatment to the endpoint of ESRD. **(D-F)** Kaplan-Meier analysis for patients with different CKD stages to the endpoint of ESRD. **(G, H)** Kaplan-Meier analysis for patients with different urine protein to the endpoint of ESRD.

Most of the patients with a high NLR underwent treatment with corticosteroids (p < 0.001), as shown in **Table 2**. Notably, the Kaplan–Meier survival curve suggested a poor renal outcome in patients with a high NLR (p < 0.001) (**Figure 3A**). Because of the influence of corticosteroids and immunosuppressive agents on the NLR, we compared the renal survival of patients with supportive treatment and those with corticosteroid or immunosuppressive agent treatment. We found that patients with a high NLR always had poor renal survival regardless of their treatment regimens (**Figures 3B, C**). Additionally, We compared the value of NLR at the time of renal biopsy and the final follow-up. We found that there was no statistical difference between two treatment groups about the change value of NLR (p=0.163) and the percentage change of NLR (p=0.141). Moreover, no statistical difference in NLR was observed in high-NLR group and low-NLR group between two treatment groups, too (**Table 4**). Furthermore, we conducted subgroup analyses with a focus on CKD stages and 24-hour urine protein. First, all patients were distributed into CKD stage 1, stage 2, and stage 3–4 groups (**Figures 3D–F**). We then divided the patients into 24-hour urine protein -proteinuria <1 and ≥1 g/day groups (**Figures 3G, H**). Our results indicated that the NLR, as a novel marker, was a risk factor for ESRD in patients with IgAN, especially patients with stage 3–4 CKD (p = 0.028) (**Figure 3F**) or 24-hour urine protein of ≥1 g/day (p < 0.001) (**Figure 3H**).

**Table 4:** The change of NLR after treatment of 966 patients.

Parameters	Treatment	The change value of NLR	P value	The percentage change of NLR	P value
All patients (n=966)	Support treatment	(-0.63, 0.44)	0.163	(-32%,25%)	0.141
	Prednisone or other immunosuppressive agents	(-0.90, 0.53)		(-26%, 22%)	
Group1 (NLR<2.67) n=582	Support treatment	(-0.20, 0.66)	0.864	(-15%, 45%)	0.814
	Prednisone or other immunosuppressive agents	(-0.27, 0.76)		(-11%, 36%)	
Group2 (NLR≥2.67) n=384	Support treatment	(-1.80, 0)	0.785	(-48%, 0)	0.614
	Prednisone or other immunosuppressive agents	(-2.10, 0)		(-52%, 0)	

NLR, Neutrophil-to-lymphocyte ratio.

The change value of NLR and the percentage change of NLR were expressed as median with interquartile range.

## Discussion

Chronic inflammation has an important effect on the onset and progression of IgAN (3). Inflammatory mediators can result in glomerular hypertension, tubulointerstitial fibrosis, and renal scarring by stimulating mesangial and endothelial glomerular cells and increasing the production while decreasing the degradation of the mesangial and endothelial extracellular matrix (10). The NLR has been recognized as a novel inflammatory marker in various diseases such as atherosclerosis (4), hepatocellular carcinoma (5), type 2 diabetes mellitus, and diabetic nephropathy (6). Among previous studies of the NLR, few have focused on IgAN. Although some interesting conclusions have been reached, previous results are controversial, possibly because of small samples and failure to assess the renal prognosis (8) and evaluate renal pathologic lesions (11).

NLR could be accessible through the neutrophil-to-lymphocyte ratio in peripheral white blood cell (WBC) count. Therefore, compared with other inflammatory markers, such as CRP and IL-6, NLR is cheaper and more widely available maker (12). Besides, NLR is a combination of the two parameters and might be a better and more stable inflammation marker than WBC count and has less probability being influenced by various physiological conditions such as dehydration (13).

The present study showed that compared with normal inflammatory factors, such as WBCs, PLTs, LYs, the PLR, and NEs, the NLR was the best predictive indicator with the best AUROC of 0.633. All patients were then divided into two groups according to the NLR cut-off of 2.67, which had a sensitivity and specificity of 64% and 62%, respectively. These results were similar to a previous study in which the NLR also had the highest AUROC (0.695) (7). Additionally, a higher NLR in patients with IgAN was associated with more severe clinical features and pathologic lesions. We found that the NLR was positively correlated with serum creatinine and 24-hour urine protein and negatively correlated with eGFR. Patients with IgAN who had a high NLR were also more vulnerable to developing hypertension. In addition, patients with IgAN who had a high NLR tended to have more severe renal pathologic lesions, especially segmental glomerulosclerosis, tubular atrophy/interstitial fibrosis, and cellular or fibrocellular crescents. To the best of our knowledge, this has not been reported in previous studies.

Our findings suggest that the NLR is an independent prognostic factor for progression to ESRD in patients with IgAN. We conducted subgroup analyses of the relationships between the NLR and CKD stages and 24-hour urine protein in patients with IgAN. First, all patients were grouped into stage 1, stage 2, and stage 3–4 CKD groups according to the CKD-EPI equation. Among patients with stage 1 and 2 CKD, those with a high or low NLR shared the same good renal outcome. This might be explained by the fact that mild renal disease progresses slowly, especially within a short follow-up period (14). Among patients with stage 3–4 CKD, a significant difference was found between the high and low NLR groups, indicating that the NLR has a greater influence in patients with stage 3–4 CKD. Similarly, a high NLR had better predictive value in patients with 24-hour urine protein of ≥1 g/day than <1 g/day. Notably, these points have not been elucidated in previous studies of patients with IgAN.

Corticosteroids have a strong anti-inflammatory effect and may influence the NLR. Therefore, we assessed the two groups of patients with respect to corticosteroid treatment. Most of the patients with IgAN who had a high NLR tended to be treated with corticosteroids, but they still had poorer renal outcomes than patients with a low NLR. Additionally, patients with IgAN who had a high NLR always had poor renal survival regardless of whether they underwent corticosteroid therapy. Because IgAN is a multifactorial disease, many other factors likely contribute to this phenomenon, and further research is therefore necessary to verify the importance of the NLR in the treatment of patients with IgAN, especially those with stage 3–4 CKD and 24-hour urine protein of ≥1 g/day.

Oxidative stress and inflammation are associated with the progression of IgAN (12). Increasing evidence has demonstrated that mucosal infection contributes to the production of aberrant glycosylated IgA and the development of IgAN (15). Complement may then be activated on the surface of circulating IgA1-containing immune complexes, resulting in the production of C3a and C5a; these are pro-inflammatory polypeptides that largely contribute to the inflammatory responses of IgAN (16).

It was reported that increased expression of complement 3 receptors and oxidative metabolism on neutrophils from IgAN patients were suspected to be relevant to the pathogenesis of IgAN (17). Notably, neutrophils could mediate the inflammation in kidney injury by a variety of biochemical mechanisms, including the release of reactive oxygen species, myeloperoxidase and proteolytic enzymes, leading to further tissue damage and renal dysfunction (18, 19).Besides, inflammation leads to lymphocyte apoptosis, down-regulation of lymphocyte differentiation and proliferation (20). Furthermore, lymphocyte was related to physiologic stress and malnutrition (21). Malnutrition may also increase the renal disease progression. The correlation between NLR, complement and malnutrition still needs to be further more studied.

Thus, NLR could reflect the balance between inflammatory and immune response, which is accepted as a marker of chronic low-grade inflammation. In addition, inflammation is one of the most important initiators of progressive tubulointerstitial fibrosis (22).

This study had three main limitations. First, it was a retrospective study in a single hospital center. Further multicentric, randomized and controlled studies are needed to evaluate the relationship between NLR and IgAN patients. Second, the mean follow-up time of 58 months was relatively short, especially for IgAN, which is a slowly progressing disease. Besides, more detail analyses about renal pathologic lesions need to performed in further study.

## Conclusion

The NLR is a significant and independent risk factor for disease progression in patients with IgAN. More attention must be paid to patients with IgAN who have a high NLR, poor renal function, and 24-hour urine protein of ≥1 g/day.

## Data Availability Statement

The raw data supporting the conclusions of this article will be made available by the authors, without undue reservation.

## Ethics Statement

The studies involving human participants were reviewed and approved by the Ethical Committee of West China Hospital of Sichuan University (2019-33). The patients/participants provided their written informed consent to participate in this study. Written informed consent was obtained from the individual(s) for the publication of any potentially identifiable images or data included in this article.

## Author Contributions

Research idea and study design: WQ, YT, SW, and LD. Data acquisition: WQ, SW, ZJ, and LD. Data analysis and interpretation: SW, LD, and WQ. Statistical analysis: SW and LD. Supervision: WQ and YT. Each author accepted accountability for the overall work by ensuring that questions pertaining to the accuracy or integrity of any portion of the work are appropriately investigated and resolved. All authors contributed to the article and approved the submitted version.

## Conflict of Interest

The authors declare that the research was conducted in the absence of any commercial or financial relationships that could be construed as a potential conflict of interest.

## Publisher’s Note

All claims expressed in this article are solely those of the authors and do not necessarily represent those of their affiliated organizations, or those of the publisher, the editors and the reviewers. Any product that may be evaluated in this article, or claim that may be made by its manufacturer, is not guaranteed or endorsed by the publisher.
